# Eco-Friendly Modified Fabrication of poly (lactic- *co*-glycolic acid) (PLGA) Nanoparticles from a Novel Utilized date seed Extract with Potent Antibacterial Properties isolated from Osteomyelitis patients.

**DOI:** 10.12688/f1000research.173692.1

**Published:** 2026-01-29

**Authors:** Elaf Sameer, Suzan Saadi Hussain, Raad S. Sabry, Saba Talib Hashim

**Affiliations:** 1 Biology, Mustansiriyah University Department of Biology, Baghdad, Baghdad Governorate, Iraq; 2 Physics, Mustansiriyah University Department of Physics, Baghdad, Baghdad Governorate, Iraq; 3 Biology, Mustansiriyah University Department of Biology, Baghdad, Baghdad Governorate, Iraq

**Keywords:** Osteomyelitis, PLGA nanoparticle, date seed extract, antimicrobial activity

## Abstract

Green nanotechnology presents an opportunity in management of osteomyelitis, whereby environmental friendly procedures are used to provide nanoparticles that are of minimal toxicity. Green nanomaterials have the potential of effectively delivering antibiotics to the infected bone sites by incorporation of biocompatible materials to improve the efficiency of the delivery as well as reducing side effects. This sustainable treatment does not only provide safer treatment but also enhances faster healing and curbs infection spread that provides a more viable and environmentally friendly solution in managing osteomyelitis.

This study is the first to synthesize poly lactic- *co*-glycolic acid (PLGA) nanoparticles by using *Phoenix dactylifera L.* (Zahidi date) seeds extract as a capping agent in a green approach. 120 osteomyelitis specimens were collected; the nanoparticles were fabricated via a modified double emulsion solvent evaporation/diffusion technique followed by lyophilization. Characterization via X-ray diffraction (XRD), Fourier transform infrared (FTIR) spectroscopy, and field emission scanning electron microscopy (FE-SEM), Antimicrobial activity and minimum inhibitory concentration of green-synthesized PLGA nanoparticles was determined. 40% of the specimens contained *Klebsiella pneumoniae* and *Pseudomonas aeruginosa*; 20%, *Staphylococcus aureus*; 10%, *Proteus mirabilis*; 10%, *Enterococcus faecium*; and 20%, different Gram-negative bacilli. PLGA nanoparticles do not exhibit considerable crystallization using the date seed extract in XRD analysis, FTIR spectroscopic analysis of PLGA nanoparticles reveals absorption bands characteristic of the polymer’s chemical structure and indicates the presence of functional groups from both PLGA and the bioactive constituents of the date pit extract. (FE-SEM) confirmed the formation of uniformly sized spherical particles (23–33 nm) with an amorphous polymeric structure and maintaining the inherent chemical integrity of PLGA. Importantly, the incorporation of bioactive phytochemicals from the date seed extract was confirmed, indicating their participation in the functional activity of the nanoparticles. Antimicrobial activities determined using the broth microdilution method revealed high inhibition against *Klebsiella pneumoniae* (MIC: 75–125 μg/mL) and *Pseudomonas aeruginosa* (MIC: 100–125 μg/mL). The findings highlight the significance of incorporating agro-industrial waste into the sustainable production of bioactive nanocarriers. The developed PLGA nanoparticles constitute an efficient carrier system with high antibacterial activity and represent a novel therapeutic strategy for treating osteomyelitis.

## 1. Introduction

Osteomyelitis is a condition of bone infection and it is one of the most challenging diseases that can be diagnosed and treated within the modern clinical practice. Current clues to its pathophysiology have shown very advanced interactions of host-pathogen especially in infections by biofilmforming *Staphylococcus aureus.* ^1^ Modern schemes support the differences in infectious diseas, transmission and vulnerability of the hosts to the clinical course. of illnesses and associated outcomes. ^2^ Acute hematogenous osteomyelitis, very frequent in children, is often due to *Streptococcus pyogenes* and *Kingella kingae*, adults experience infections of *Staphylococcus aureus* (including MRSA), *Enterococcus* and *Streptococcus agalactiae* . ^3^ Spinal osteomyelitis, often given in elderly patients is normally caused by *Escherichia coli*, *Klebsiella pneumoniae*, and *Mycobacterium tuberculosis.* ^4, 5^ Post-surgical and post-traumatic osteomyelitis frequently involve species such as *Pseudomonas aeruginosa* and *Proteus mirabilis* and anaerobic bacteria such as *Peptostreptococcus* and *Bacteroides fragilis.* ^6^

Osteomyelitis continues to present a significant global concern, with growing antimicrobial resistance issues and rising incidence levels among the elderly and those diagnosed with immunosuppression, diabetes, and peripheral vascular diseases. ^7^ In this regard, nanotechnology offers improved platforms for drug delivery and antimicrobial therapy, transforming the biomedical sciences. Among the many developed nanomaterials, poly (lactic- *co*-glycolic acid) (PLGA) nanoparticles incorporate biocompatibility together with a high and flexible drug-carrying capacity and are noted for their controlled drug release, biodegradability, and high safety profile. ^8^

The application of plant extracts as capping agents of the production of nanoparticles minimizes the application of dangerous chemicals as well enhancing the bioactivity of the nanoparticles. ^9^ Notably, the high date pit extracts polyphenol and antioxidants compounds levels provide the biologic activity and stability of nanoparticles. ^10^ These green techniques of synthesis enhance uniformity and size distributions of nanoparticles., which are important parameters enhancing cellular uptake and therapeutic efficacy. ^11, 12^ Besides that, recent discovery on the green preparation of magnesium oxide nanoparticles (MgO NPs) by using Plant-based extracts ( *Allium sativum*) have been shown to possess the potential ways to be green, simple and efficient plans on how to serve preparation of nanomaterials with excellent antibacterial properties. ^13^

Recent researches have shown that PLGA nanoparticles can encapsulate antimicrobial compounds and efficiently deliver these compounds to the infected bone tissue, thereby significantly decreasing the bacterial occurance and improving bone healing process. ^14, 15^ While the green synthesis of PLGA nanoparticles using date seed extracts holds significant promise, addressing the challenges related to scalability and reproducibility is essential for their successful transition to industrial applications. Ongoing research and development efforts are needed to refine synthesis methods, optimize production processes, and ensure the safety and efficacy of these nanoparticles in large-scale applications.

This research proposes an environmentally friendly method for producing PLGA nanoparticles using *Phoenix dactylifera L.* (Zahidi date) seeds extractx, ^33^ given its high polyphenol and antioxidant content, which contributes to the biological activity and stability of nanoparticles. In addition, the study aims to characterize the physical properties and evaluate the biomedical potential of PLGA nanoparticles. By integrating the advantages of biodegradable polymer carriers with the inherent bioactivity of natural plant molecules, this approach is likely to yield an efficient and sustainable platform for the management of osteomyelitis and other difficult-to-treat infections.

## 2. Materials and methods

### 2.1 Materials

Reagents and materials—including PLGA (75:25, molecular weight [MW]) = (10,000 g/mol) (GLOPBIO), ethanol (Catalogue No. 100983/Merck Millipore), dichloromethane (DCM) (Catalogue No. 106050/Merck Millipore), acetone (ACE) (Catalogue No. 423240010/Thermo Scientific), *Phoenix dactylifera L.* (Zahidi date) seeds (from local Baghdad/Iraq palm trees), polyvinyl alcohol (PVA) (Catalogue No. 114266/Merck Millipore), brain heart broth (Catalogue No. CM1135B/Thermo Scientific), and dimethyl sulfoxide (DMSO) (Catalogue No. 472301/Sigma-Aldrich).

### 2.2 Sample collection

A total of 120 osteomyelitis specimens were collected from Al-Kindy teaching hospital, Al Wasiti hospital, and Baghdad teaching hospital in Baghdad city. The specimens were taken by blood, bone lesion swabs, and deep lesion aspiration. All specimens were transported to the laboratory and inoculated onto blood agar and MacConkey agar at 37°C overnight under aerobic conditions.

### 2.3 Biosynthesis of PLGA nanoparticles from date seed extract

The seeds of date fruits were removed manually, washed with deionized water, and left to dry at room temperature. The date seeds were then ground with an electric grinder apparatus, and (1 g) of the date seed powder was dissolved in (10 ml) of an ethanol/water mixture (8:2) and left to stand overnight. The PLGA nanoparticles were fabricated via the modified double emulsion (water/oil) solvent evaporation/diffusion method of. ^16^ In brief, 0.2 mg of PLGA (75:25), with a MW of (10,000) g/mol, was dissolved in 10 mL of a DCM/ACE mixture (8:2) containing the emulsifier Tween 80 (5% v/v). The date seed solution was filtered using filter paper, and 2 mL of the date seed extract was added to the polymer solution flask and homogenized via stirring for 2 min at a speed of 50%. The initial water/oil emulsion was then added to (40 mL) of PVA (0.5%) with mixing for 2 min to obtain a stable double emulsion (W/O/W). The resulting emulsion was added slowly to 60 mL of an aqueous PVA solution (0.3%) as the surfactant, and the pH was adjusted to 9–10 under steady magnetic stirring overnight to solidify the nanoparticles. Thereafter, the emulsion was sonicated in an ice bath for 10 min (in 2-min intervals with 1-min pauses). The PLGA nanoparticles were collected via ultracentrifugation at (17,000) rpm and three times washing with distilled water, after the first washing, the nanoparticle emulsion was sonicated in an ice bath for 5 min, followed by the next two washings. Finally, the products were lyophilized and kept at 4°C. The green synthesis of the PLGA nanoparticles is depicted in Figure 1.

**Figure 1.**
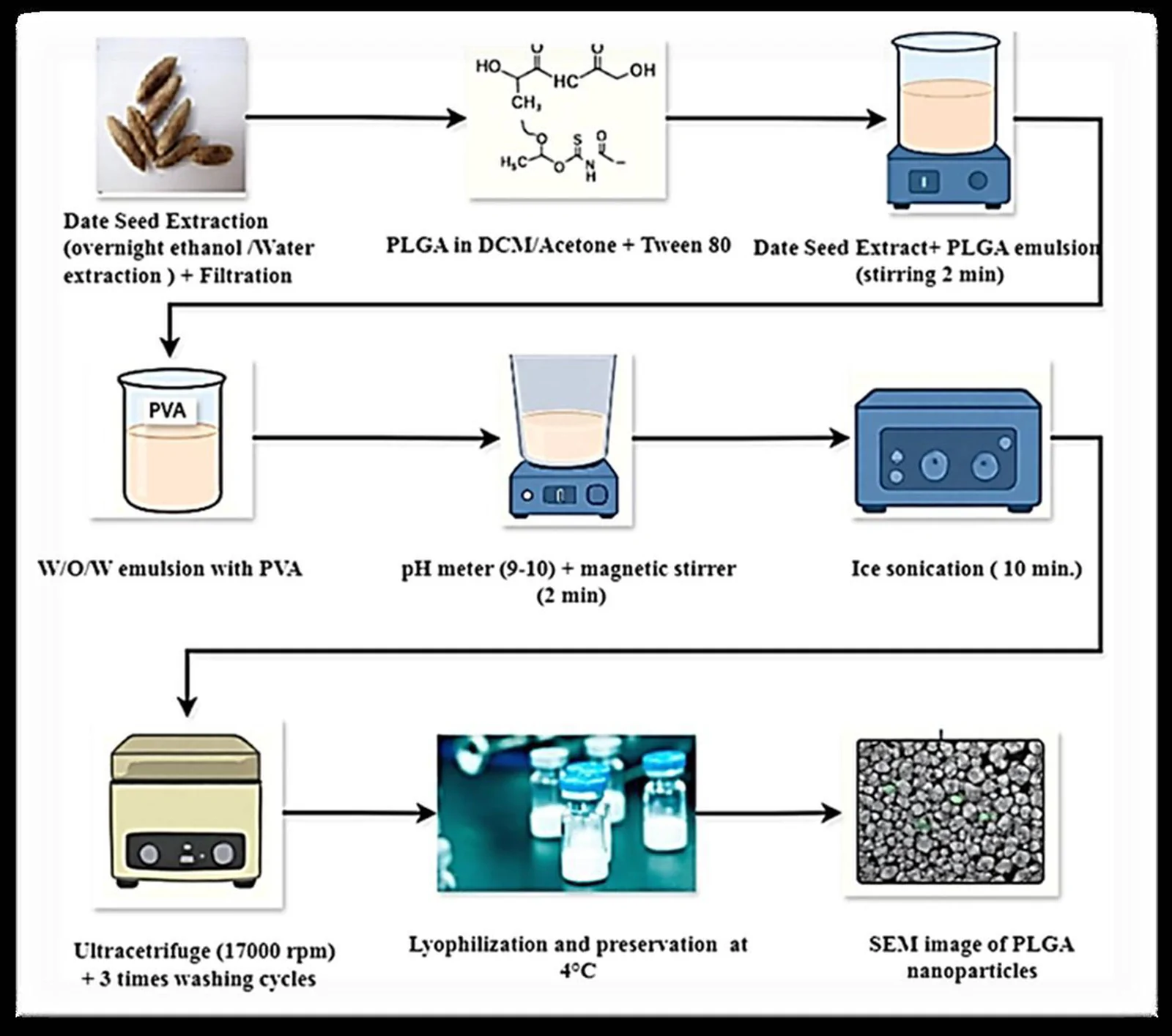
Schematic of PLGA NPs. Preparation process by date seed extract.

### 2.4 Characterization of biosynthesized PLGA nanoparticles

The PLGA nanoparticles were characterized via several techniques, including field emission scanning electron microscopy (FE-SEM), X-ray diffraction (XRD), and Fourier transform infrared (FTIR) spectroscopy.

### 2.5 Antimicrobial activity of green-synthesized PLGA nanoparticles

*Pseudomonas aeruginosa* and *Klebsiella pneumoniae* were utilized in this study, with both strains grown aerobically at 37°C overnight in brain heart broth. The bacterial suspensions were adjusted to a 0.5 McFarland standard turbidity of ~1 × 10 ^6^ CFU/mL for the assay. The minimum inhibitory concentration (MIC) of the PLGA nanoparticles was evaluated via a standard broth microdilution technique in a 96-well microtiter plate. A PLGA nanoparticle stock solution was prepared by dissolving (10 mg) of the nanoparticles in (10 mL) of DMSO to yield a final concentration (1,000 μg/mL); solutions of different concentrations (25, 50, 75, 100, 125, 150, 175, 200, 225, 250, 275, 300, 325, 350, 375, and 400 μg/mL) were then prepared from stock solution. Each well was supplied with 100 μL of brain heart broth, 100 μL of the nanoparticle suspension, and 2 μL of bacterial broth. A positive control containing the bacterial broth (with no nanoparticles) and a negative control containing only the brain heart broth were used. The plates were incubated in aerobic conditions at 37°C for 18–24 h.

### 2.6 Determination of MIC of PLGA nanoparticles

Following incubation, the MIC—the minimum concentration of PLGA nanoparticles resulting in ≥90% inhibition of bacterial growth—was assessed by measuring the optical density at 600 nm (OD _600_) using a microplate reader. ^17^

## 3. Results

### 3.1 Identification of pathogenic bacteria isolated from osteomyelitis specimens

A total of 120 osteomyelitis specimens were analyzed: approximately 40% of the specimens contained *Klebsiella pneumoniae* and *Pseudomonas aeruginosa*; 20%, *Staphylococcus aureus*; 10%, *Proteus mirabilis*; 10%, *Enterococcus faecium*; and 20%, different Gram-negative bacilli. All isolates were identified by using biochemical testing and Vitek 2 Compact system apparatus.

### 3.2 Characterization of biosynthesized PLGA nanoparticle

**3.2.1 X-ray diffraction (XRD)**

Regarding the XRD analysis, the green-synthesized PLGA nanoparticles do not exhibit considerable crystallization or crystalline impurities using the date seed extract, displaying characteristic broad diffraction peaks. The results also reveal the presence of a mainly amorphous polymeric state ( Figure 2). ^34^ This agrees with the native semicrystalline nature of PLGA, arising from the random configurations of lactic and glycolic acid units in the copolymer, which lead to a low degree of crystallinity and mainly amorphous domains. In an XRD plot, the presence of broad peaks at low 2θ angles typically indicates that PLGA is either amorphous or semi-crystalline. These broad peaks represent the distance between polymer layers or irregular molecular arrangements on a nanometric scale. ^18^

**Figure 2.**
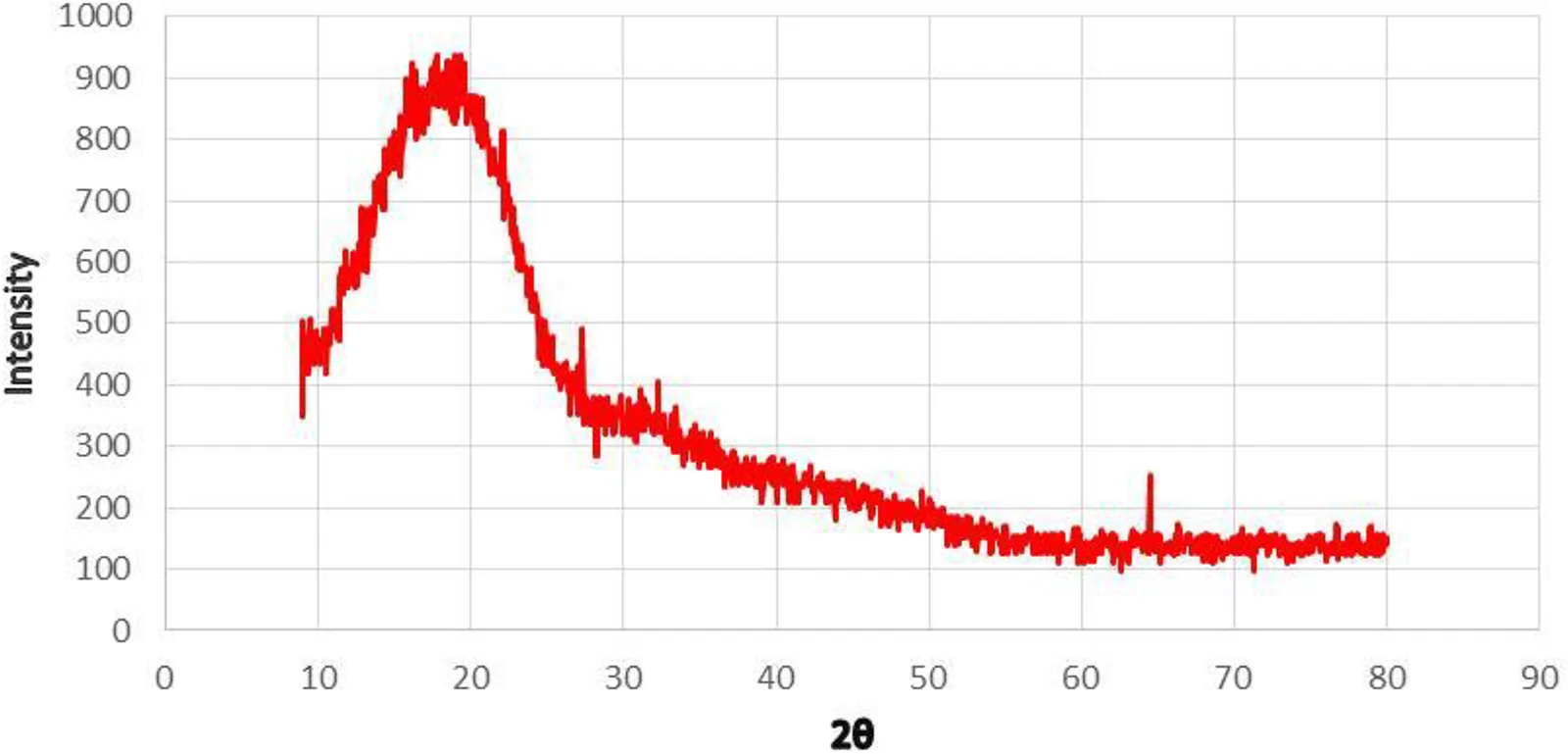
XRD of date seed extract-based PLGA nanoparticles.

**3.2.2 FTIR spectroscopy**

FTIR spectroscopic analysis of the green-synthesized PLGA nanoparticles reveals absorption bands characteristic of the polymer’s chemical structure and indicates the presence of functional groups from both PLGA and the bioactive constituents of the date pit extract. ^34^ The major peaks include a broad absorption band at 3432 cm ^−1^, corresponding to O–H stretching vibrations; this is generally due to hydroxyl groups ( Figure 3), possibly arising from the presence of phenolic compounds or moisture in the date pit extract. ^14^

**Figure 3.**
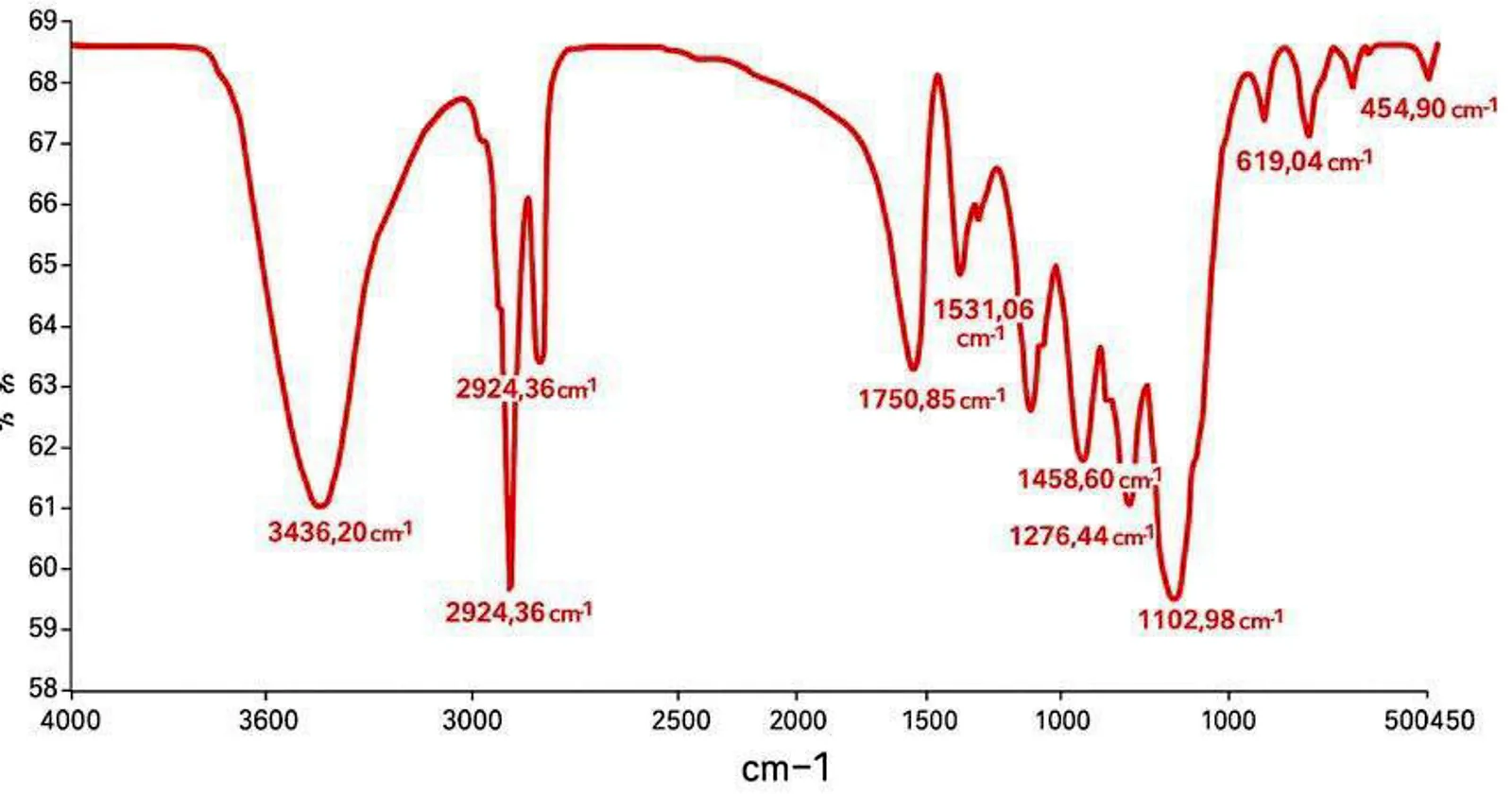
FTIR spectroscopy of the green synthesized PLGA nanoparticles using date seed extract.

The peaks at 2956 and 2924 cm ^−1^ are associated with the symmetric and asymmetric stretches of aliphatic C–H chains, corresponding to the polymer backbone of PLGA. ^18^ Strong absorption at 1734 cm ^−1^ indicates ester carbonyl (C=O) stretching vibrations characteristic of the polyester structure of PLGA. ^8^ The appearance of this peak confirms that the integrity of the polymer backbone is maintained after synthesis.

Further bands at 1464 and 1384 cm ^−1^ are assigned to CH _3_ deformation modes, while the band at (1110 cm ^−1)^ is assigned to (C–O–C) stretching vibrations, representative of ester linkages. ^20^ The fingerprint region (below 1000 cm ^−1^) includes bands at 875 and 799 cm ^−1^, which could be attributed to the polymer chain vibrations and the phytochemical moieties introduced by the date seed extract.

**3.2.3 Field emission scanning electron microscopy (FE-SEM)**

The FE-SEM micrograph of the synthesized PLGA nanoparticles reveals a spherical shape and uniformly distributed particle sizes (23–33 nm), as shown in Figure 4. A uniform particle size distribution is significant in biomedical implementations such as drug delivery systems and antimicrobial therapies, where uniformity in particle size and shape impacts drug cellular uptake and bioavailability. ^8, 23^

**Figure 4.**
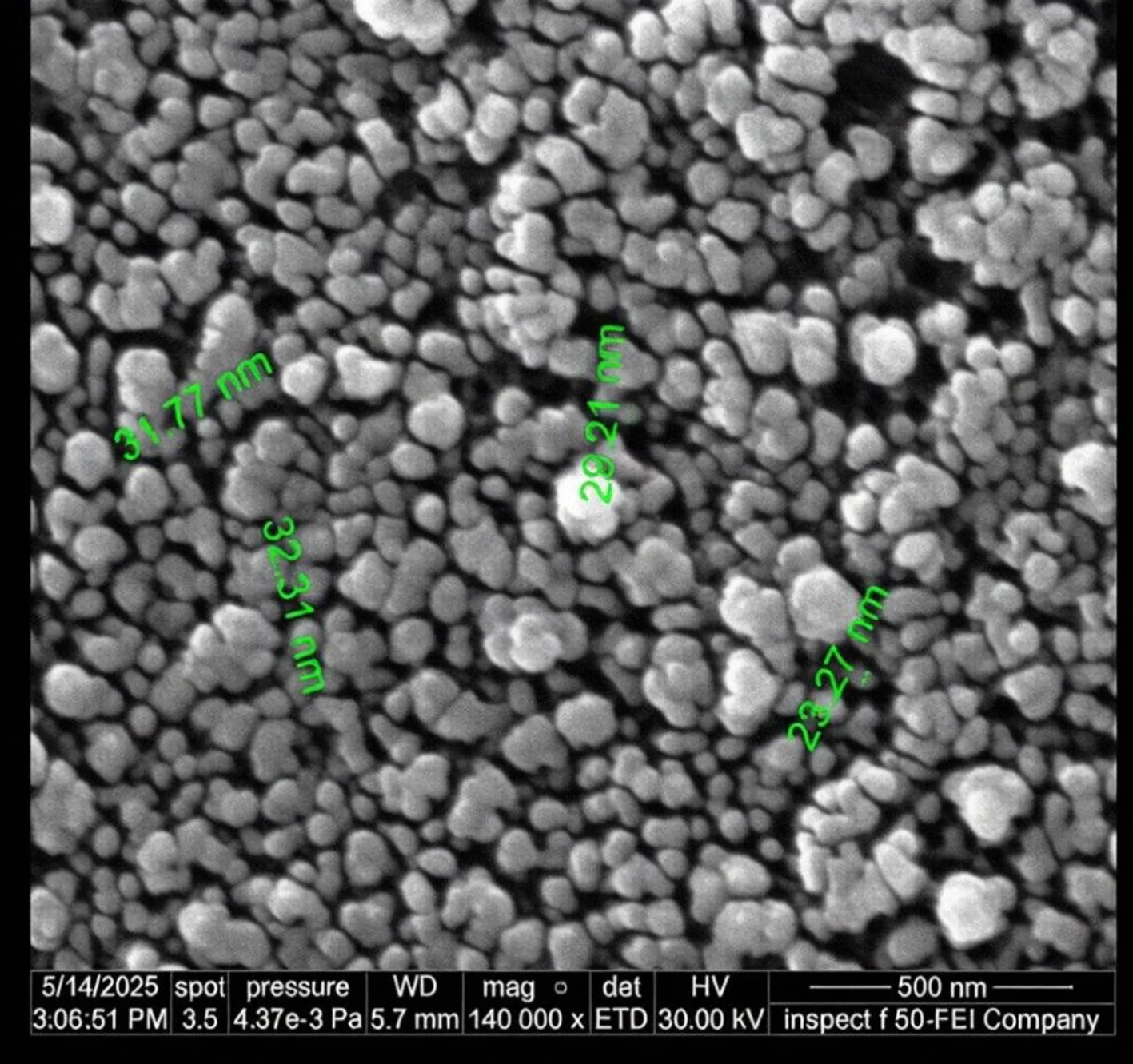
FESEM image of date pits extract- green synthesized PLGA nanoparticles.

**3.2.4 Determination of MIC of PLGA nanoparticles via microdilution**

A range of concentrations (25–400 μg/mL) was used in determining the MIC of the PLGA nanoparticles. The MIC is (100 μg/mL) for eight isolates, (125 μg/mL) for 10 isolates, and 75 μg/mL for two isolates of *Klebsiella pneumoniae.* On the other hand, the MIC values are (100 μg/mL) for six isolates and (125 μg/mL) for 14 isolates of *Pseudomonas aeruginosa* ( Table 1 and Figure 5).

**Table 1.** MIC of PLGA nanoparticles with different concentration.

PLGA NPs. concentrations (μg/ml)	No. of isolates	
	*Klebsiella pneumoniae*	*Pseudomonas aeroginosa*
75	2	∕
100	8	6
125	10	14

**Figure 5.**
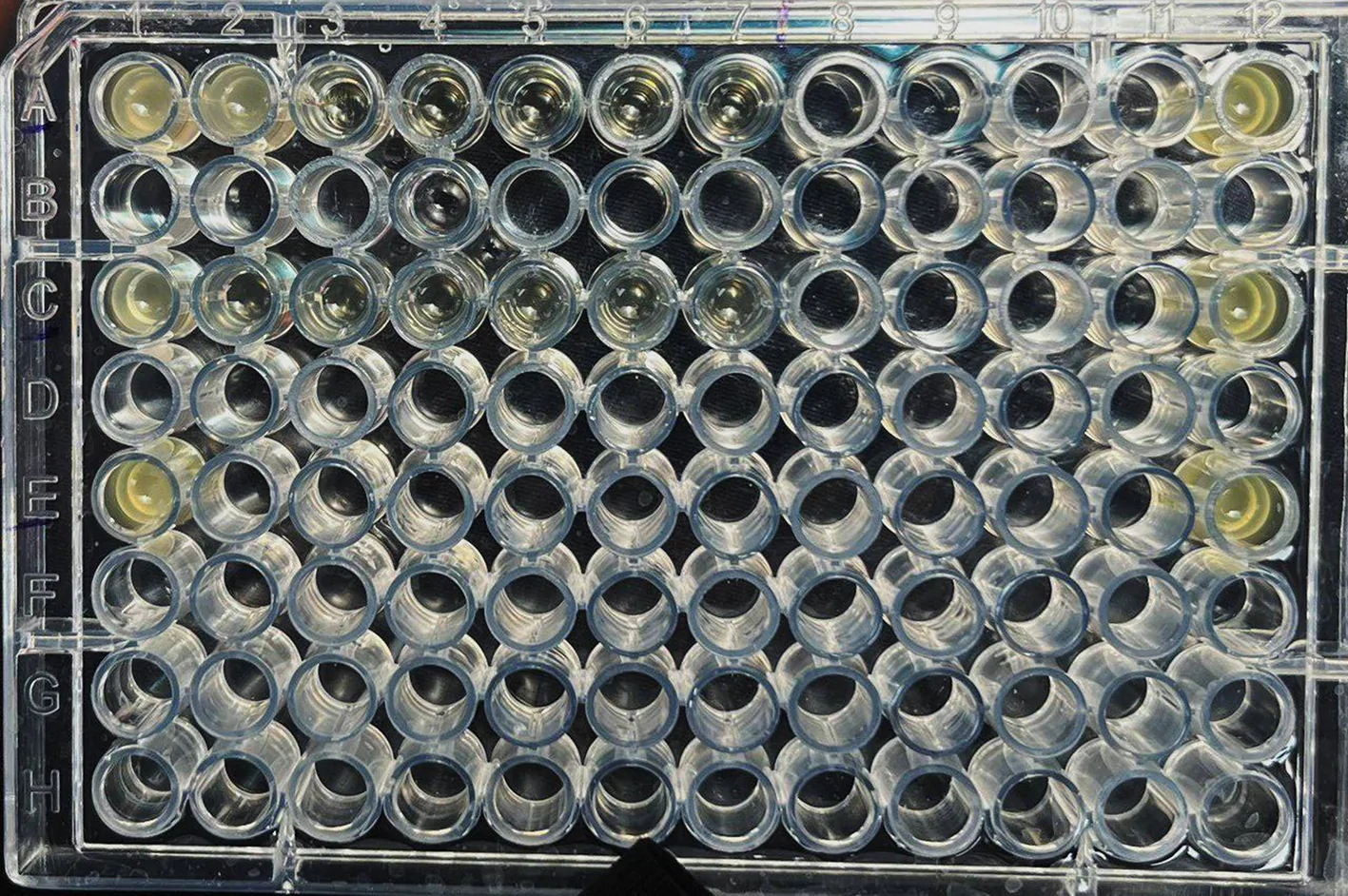
Determination of Minimum inhibitory concentrations (MICs) of PLGA nanoparticles, in a 96-well microtiter plate.

## 4. Discussion

### 4.1 Characterization of biosynthesized PLGA nanoparticle

**4.1.1 X-ray diffraction (XRD)**

The resultant amorphous nature of the nanoparticles is beneficial in biomedical applications, especially in drug delivery, since amorphous PLGA supports faster and more controlled biodegradation and drug release kinetics compared to its crystalline counterparts. ^8^ Moreover, bioactive phytochemicals present in date pit extract can interact with the polymer chains and disrupt the crystallinity to an even greater degree, which can stabilize and functionalize the nanoparticles in the process. ^9^

The XRD patterns herein are consistent with recent studies in which PLGA nanoparticles were prepared via traditional techniques such as nanoprecipitation and solvent evaporation; these likely lead to diffuse, broad peaks, indicating amorphous PLGA structures, ^19, 20^ for instance the study of ^20^ showed that the crystallinity of PLGA nanoparticles is typically low, which supports the broad XRD peaks observed herein.

Green synthesis procedures employing plant extracts, such as the current utilization of the date pit extract, have increasingly been identified as maintaining or augmenting this amorphous nature, incorporating, in the process, functional bioactive groups capable of dictating particle behavior. ^10^

The integration of phytochemical interactions of the date pit extract with the amorphous nature of PLGA enhances the biocompatibility and biodegradability of the nanoparticles, which is significant in terms of antimicrobial activity and drug delivery. ^21^ This is further corroborated in recent reports where bio-extracts not only act as reducing agents in green synthesis but also enhance functional performance via the structural modulation of polymer matrices. ^12, 22^

**4.1.2 FTIR spectroscopy**

The FTIR spectral profile of PLGA nanoparticles is in good agreement with that previously recorded in the literature for conventional and green-synthesized PLGA nanoparticles. One study noted the same preservation of characteristic PLGA bands in plant extract nanoparticles, indicating a well-maintained polymeric structure and good inclusion of bioactive components without substantial chemical changes. ^10^

Furthermore, the wide hydroxyl bands confirm the physical adsorption of the phenolic molecules from the date seed extract onto the surface of the nanoparticles, possibly contributing to an improvement in their functional properties, such as their antimicrobial or antioxidant activity. ^21^ These types of interactions are consistent with the findings of another reseach findings which reported that bio-extract components are likely to form hydrogen bonds or other types of interactions with polymer matrices that are capable of controlling the surface chemistry of nanoparticles. ^12^

Finally, the FTIR analysis confirms that the synthesized PLGA nanoparticles retain the polymer chemical structure and incorporate bioactive functionalities. The findings are consistent with recent literature studies on the green synthesis of nanoparticles, which have highlighted the dual benefit of polymer biocompatibility and natural extract bioactivity for biomedical applications.

**4.1.3 Field emission scanning electron microscopy (FE-SEM)**

The plant-based bio-reducing and stabilizing nature of the date seed extract might have induced nucleation and hindered particle agglomeration, promoting isotropic growth and restraining the particle size. The use of phytochemicals in the present synthesis is also in agreement with the relatively new trend of environmental “green” nanoparticle synthesis, which limits the use of harmful chemicals and increases biocompatibility. ^10^

The sizes of the nanoparticles synthesized herein (23–33 nm) are smaller than those reported in other works for PLGA nanoparticles prepared via classical procedures (ranging between 50 and 200 nm) with a broader polydispersity. ^19, 24^ For instance, previous study developed 40–70-nm PLGA particles via solvent evaporation, with no aggregation observed in the PLGA green synthesis. ^11^ In addition, similar particle size homogeneity (~30 nm) reported using plant extract-based reduction agents, indicative of the efficiency of biogenic synthesis toward achieving controlled nanostructures. ^9^

Spherical particle shapes and smaller particle sizes enhance the surface area, with cell interactions providing more efficient drug delivery and release. ^10, 21^ Furthermore, the morphology of the particles can promote their antibacterial effect, as particles of this size can more easily pass through the bacterial cell membrane and biofilms. ^12^

Overall, the FE-SEM images confirm that the synthesis of PLGA nanoparticles using extract of date seed is an eco-friendly and reliable method, enhancing the physicochemical properties of the nanoparticles compared to conventional chemical methods. This synthetic method appears notably promising in terms of offering biocompatibility and precision.

**4.1.4 Determination of MIC of PLGA nanoparticles via microdilution**

The MIC analysis of the PLGA nanoparticles against *Klebsiella pneumoniae* and *Pseudomonas aeruginosa* revealed encouraging antimicrobial activities. The MIC values for *K. pneumoniae* range from 75 to 125 μg/mL, with most isolates showing an MIC of 125 μg/mL. Thus, these nanoparticles could effectively inhibit the growth of *K. pneumoniae*, an opportunistic pathogen responsible for pneumonia and urinary tract infections (UTIs) within clinical centers. ^25^ The broad range of MICs signals differences in the susceptibility of *K. pneumoniae* isolates, attributable to strain-specific traits, resistance mechanisms, or biofilm formation abilities. ^26^

*Pseudomonas aeruginosa* is the most famous example of a multidrug-resistant organism able to cause chronic infections particularly in weakened hosts, ^24^ is slightly different in the profile of susceptibility specifically MIC of 125 μg/mL is obtained with 14 strains and 100 μg/mL is found to be inhibited in six. This increase in MIC proportionately of *P. aeruginosa* could be attributed to the high resistance introduced by efflux pumps and the beta-lactamase secretion system. ^27^ The results are in line with earlier reports on green synthesis of nanoparticles on plants. Our date seed extract-generated PLGA nanoparticles have a homogeneous ultrastructure and good antibacterial ability, equivalent to the Cr _2_O _3_ nanoparticles of, ^28^ that also had demonstrable microbial inhibition with the photocatalyzing of dyes. The comparison thus brings out the versatility and functional benefit of the biodegradable polymeric carriers conjugated with bioactive compounds of plants. Prior work is also concurred with our results, which suggests the possibility of PLGA nanoparticles to enhance antimicrobial action given their biodegradability and biocompatibility. ^29^ PLGA nanoparticles outperform traditional common antibiotics by enhancing drug bioavailability and targeting via controlled drug release and longer bacterial interactions, enabling g drug efficiency at lower concentrations than free drug forms. ^30^ Antibacterial mechanism of PLGA nanoparticles can show by physically interaction with bacterial cell membranes belong to their small in size and surface properties. The nanoparticles may adhere or adsorb to the bacterial cell wall, causing physical disruption or damage of the cell membrane, which leads to bacterial death. PLGA nanoparticles can also cause immune system activation leading to an enhanced body immune response against bacterial infections. This can further support the antibacterial activity. ^31^ Other studies suggest that PLGA nanoparticles can generate reactive oxygen species (ROS) when they come into contact with bacteria. These ROS can damage essential components of bacterial cell like DNA, proteins, and lipids, leading to certain bacterial death. ^32^

## 5. Conclusions

This work documents the novel green synthesis of PLGA nanoparticles using date seed extract, a readily accessible agro-industrial waste biomass material. By employing this extract as a capping agent, waste biomass was successfully transformed into a high-value nanomaterial platform. The resultant nanoparticles exhibited a significantly uniform morphology, with ultra-small particle sizes and excellent colloidal stability. Spectroscopic and structural analyses confirmed the presence of phenolic compounds from the date seed extract in the PLGA matrix. These phytoconstituents have medicinal value and significantly enhanced the antimicrobial activity of the nanoparticles, as demonstrated by their strong inhibitory activity against *Klebsiella pneumoniae* and *Pseudomonas aeruginosa* isolated from osteomyelitis patients. This represents scientific progress in developing eco-friendly treatments for osteomyelitis. The immersion of plant residues in biodegradable PLGA introduces a novel nanosystem which highlights the green potential of waste-based nanotechnology in combating the global issue of antimicrobial resistance.

## Ethical consideration

This research paper was conducted in accordance with the ethical standards outlined in the Declaration of Helsinki regarding human participation in research. The Ethics Committee granted its ethical approval, ensuring that all ethical considerations for conducting research involving human subjects were met. The objectives and data collection methods of the study were fully explained to all participants. Given the sensitive nature of the patient population, which included individuals experiencing physical and emotional distress due to their medical condition, verbal informed consent was deemed more appropriate. This approach was chosen to ensure that patients were not further burdened by the formalities of written consent, thus reducing any additional stress and ensuring that their participation remained voluntary and comfortable. The collage of science/Al Mustansiriyah University Ethics committee of Biology gave the ethical approval (Ref.: BCSMU/14025/00064M dated May 21, 3, 2025).

## Data Availability

Repository name: FTIR and XRD dataset for [Eco-Friendly Modified Fabrication of poly (lactic-co-glycolic acid) (PLGA) Nanoparticles from a Novel Utilized Date Seed Extract with Potent Antibacterial Properties Isolated from Osteomyelitis Patients]. [ https://doi.org/10.5281/zenodo.18208346] ^34^ This project contains the following underlying data: FTIR_spectrum.raw (raw FTIR spectral data) XRD_data.raw (raw X-ray diffraction data) Data are available under the Creative Commons Attribution 4.0 International license
