# Supplementary material for: Eco-Friendly Modified Fabrication of poly (lactic- co-glycolic acid) (PLGA) Nanoparticles from a Novel Utilized date seed Extract with Potent Antibacterial Properties isolated from Osteomyelitis patients
Source: F1000Res. 2026 Oct 2;15:152. Originally published 2026 Jan 29. [Version 2] doi: 10.12688/f1000research.173692.2 (PMC13650325; doi:10.12688/f1000research.173692.2)
Supplement: Peer review history

# Reviewer report: Reviewer response for version 2

**DOI:** 10.5256/f1000research.210698.r527312

**Published:** 2026-10-09

**Authors:** Priya Patel

**Affiliations:** 1 Saurashtra University, Rajkot, Gujarat, India

1. In introduction section lacking about the information of green nanotechnology
2. Rational is not clear towards the readers
3. Rationale for selecting date seed extract should be strengthened.
4. The experimental procedure should be thoroughly rewritten with sufficient step-by-step details, including all processing conditions and parameters. The make, model, and specifications of all instruments used should also be clearly provided to ensure reproducibility of the study.
5. Characterization of the prepared nanoparticles should be comprehensive.
6. Source and preparation of the date seed extract require detailed description
7. Isolation of bacterial strains from osteomyelitis patients should be described in sufficient detail.
8. Antibacterial activity requires stronger scientific validation. The authors should clearly report the bacterial strains tested, inoculum standardization, experimental controls, replicates, and statistical analysis. MIC/MBC or other quantitative antibacterial assays should be considered where appropriate.
9. The antibacterial contribution of the date seed extract and PLGA nanoparticles should be differentiated.
10. The findings should not merely be presented descriptively; the authors should scientifically explain the observed changes in particle size, morphology, surface characteristics, and antibacterial activity and correlate these findings with the formulation mechanism.
11. Findings are mainly presented descriptively, with insufficient scientific interpretation and correlation with the objectives of the study.
12. Antibacterial results should be discussed comparatively, including free date seed extract, blank PLGA nanoparticles, and date seed extract-modified/loaded PLGA nanoparticles, to establish the actual contribution of nanoparticle formulation.
13. The proposed mechanism of antibacterial action should be discussed carefully.
14. Discussion should be strengthened by comparing the present findings with previously published PLGA-based and plant-extract-mediated nanoparticle systems, highlighting similarities, differences, and the specific advancement of the present study.
15. Conclusion is too general it should be specific.
16. Several sentences should be grammatically corrected, and the terminology, abbreviations, units, and nomenclature should be made consistent throughout the manuscript.

Is the work clearly and accurately presented and does it cite the current literature?

No

If applicable, is the statistical analysis and its interpretation appropriate?

Partly

Are all the source data underlying the results available to ensure full reproducibility?

No

Is the study design appropriate and is the work technically sound?

No

Are the conclusions drawn adequately supported by the results?

No

Are sufficient details of methods and analysis provided to allow replication by others?

No

Reviewer Expertise:

NDDS, Nannoparticultae DDS, Vaginal DDS, Microparticulate DDS, Dissolution enhancement

I confirm that I have read this submission and believe that I have an appropriate level of expertise to confirm that it is of an acceptable scientific standard, however I have significant reservations, as outlined above.

# Reviewer report: Reviewer response for version 2

**DOI:** 10.5256/f1000research.210698.r526675

**Published:** 2026-10-07

**Authors:** Rawa Abdul Redha Aziz

**Affiliations:** 1 Al-karkh University of Science, Baghdad, Iraq

The manuscript is now satisfying and ready to be indexed. All my comments are answered by the author.

Is the work clearly and accurately presented and does it cite the current literature?

Yes

If applicable, is the statistical analysis and its interpretation appropriate?

Yes

Are all the source data underlying the results available to ensure full reproducibility?

Yes

Is the study design appropriate and is the work technically sound?

Yes

Are the conclusions drawn adequately supported by the results?

Yes

Are sufficient details of methods and analysis provided to allow replication by others?

Yes

Reviewer Expertise:

Microbiology

I confirm that I have read this submission and believe that I have an appropriate level of expertise to confirm that it is of an acceptable scientific standard.

# Reviewer report: Reviewer response for version 1

**DOI:** 10.5256/f1000research.191530.r510177

**Published:** 2026-09-14

**Authors:** Rawa Abdul Redha Aziz

**Affiliations:** 1 Al-karkh University of Science, Baghdad, Iraq

**Major comments**

 1. The novelty of the study should be better justified…

 2. Important nanoparticle characterization data (DLS, PDI, zeta potential) are missing…

 3. Appropriate experimental controls should be included…

 4. The statistical analysis requires further clarification…

 5. The discussion overstates the antibacterial mechanism without direct experimental evidence…

**Minor comments**

 * Correct grammatical and typographical errors.

 * Italicize bacterial species names consistently.

 * Improve figure quality.

 * Update several references with recent literature.

 The manuscript is generally well organized, and the characterization techniques (XRD, FTIR, FE-SEM) support nanoparticle formation. The work is clearly and accurately presented and it is used some of pathogens of interest among Iraqi researchers since they cause many diseases in the area. . Overall, this work is good and scientifically sound.

Is the work clearly and accurately presented and does it cite the current literature?

Yes

If applicable, is the statistical analysis and its interpretation appropriate?

Yes

Are all the source data underlying the results available to ensure full reproducibility?

Yes

Is the study design appropriate and is the work technically sound?

Yes

Are the conclusions drawn adequately supported by the results?

Yes

Are sufficient details of methods and analysis provided to allow replication by others?

Yes

Reviewer Expertise:

Microbiology

I confirm that I have read this submission and believe that I have an appropriate level of expertise to confirm that it is of an acceptable scientific standard, however I have significant reservations, as outlined above.

# Response

**Published:** 2026-09-14

**Authors:** Elaf Sameer

**Affiliations:** Biology, Mustansiriyah University Department of Biology, Baghdad, Baghdad Governorate, Iraq

**Response to Reviewer**

**Manuscript Title:**

*Eco-Friendly Modified Fabrication of Poly(lactic-co-glycolic acid) (PLGA) Nanoparticles from a Novel Utilized Date Seed Extract with Antibacterial Properties against Osteomyelitis-Associated Clinical Isolates*

**Dear Reviewer,**

 We sincerely thank the reviewer for the careful evaluation of our manuscript and for providing valuable and constructive comments. The reviewer’s suggestions have helped us improve the scientific clarity, accuracy, and interpretation of our findings.

 We have carefully revised the manuscript according to all comments. The modifications have been incorporated into the revised manuscript and highlighted for clarity. Detailed responses to each comment are provided below.

**Major Comments**

**Comment 1:**

**The novelty of the study should be better justified.**

**Response:**

 We sincerely appreciate the reviewer’s valuable comment. We agree that the novelty of the study required further clarification.

 Accordingly, the Introduction section has been revised to better explain the scientific contribution and research gap addressed by this work. Although PLGA nanoparticles have been extensively investigated for biomedical applications, the utilization of natural plant-derived extracts as functional components during PLGA nanoparticle fabrication remains relatively less explored.

 In the revised manuscript, we clarified that the novelty of the present study is based on the use of *Phoenix dactylifera L.* (Zahidi date) seed extract as a natural bioactive component during PLGA nanoparticle fabrication and the evaluation of the antibacterial activity of the developed nanoparticles against clinical osteomyelitis-associated isolates.

**Location of revision:**

 Introduction section (new highlighted paragraph).

**Comment 2:**

**Important nanoparticle characterization data (DLS, PDI, zeta potential) are missing.**

**Response:**

 We thank the reviewer for highlighting the importance of these nanoparticle characterization parameters.

 We agree that DLS, PDI, and zeta potential measurements provide valuable information regarding hydrodynamic particle size distribution, polydispersity, and surface charge characteristics. However, these analyses were not available during the experimental period due to limitations in instrument accessibility.

 Therefore, the revised manuscript has been modified to acknowledge this limitation. We have avoided unsupported statements regarding colloidal stability, hydrodynamic particle size, and surface charge properties. The interpretation of nanoparticle characteristics is now limited to the available characterization techniques, including FE-SEM, XRD, and FTIR analyses.

 A limitation statement regarding the need for additional physicochemical characterization has been added to the revised manuscript.

**Location of revision:**

 Study limitations section and related nanoparticle characterization discussion.

**Comment 3:**

**Appropriate experimental controls should be included.**

**Response:**

 We appreciate the reviewer’s important comment regarding experimental controls.

 The antibacterial assay included a bacterial growth control containing bacterial broth without nanoparticle treatment and a sterility control containing culture medium without bacterial inoculation. In the revised manuscript, the role of these controls has been clarified to demonstrate that the observed bacterial inhibition was associated with nanoparticle exposure.

 We also acknowledge that additional comparative controls, including PLGA nanoparticles without date seed extract, date seed extract alone, and standard antibacterial agents, would provide further information regarding the contribution of each component to the observed antibacterial activity. This point has been added as a limitation of the current study.

**Location of revision:**

 Materials and Methods section – Antibacterial activity assay; Study limitations section.

**Comment 4:**

**The statistical analysis requires further clarification.**

**Response:**

 We appreciate the reviewer’s comment regarding statistical analysis.

 The aim of the antibacterial evaluation in the present study was to determine the minimum inhibitory concentration (MIC) values for individual clinical isolates rather than to compare different experimental groups.

 Therefore, the obtained MIC values were interpreted based on the minimum nanoparticle concentration required to inhibit bacterial growth. Since the experimental design was not based on comparative treatment groups, inferential statistical analysis was not performed.

 This clarification has been added to the revised manuscript to provide a more transparent description of the data interpretation approach.

**Location of revision:**

 MIC determination section.

**Comment 5:**

**The discussion overstates the antibacterial mechanism without direct experimental evidence.**

**Response:**

 We sincerely thank the reviewer for this important observation.

 We agree that the antibacterial mechanisms proposed in the original manuscript required more cautious interpretation because direct mechanistic assays were not performed in the present study.

 Accordingly, the Discussion section has been revised. The proposed mechanisms, including nanoparticle interaction with bacterial surfaces and oxidative stress-related effects, are now presented as possible explanations based on previous literature rather than confirmed mechanisms demonstrated by the current experimental results.

 The revised discussion now states that interactions between nanoparticle surfaces and bacterial cells may influence membrane-associated processes, while oxidative stress-related mechanisms require further experimental confirmation.

 Furthermore, SEM-based investigation of bacterial morphological changes following PLGA nanoparticle exposure is currently being conducted as part of our ongoing research to further understand nanoparticle–bacteria interactions.

 The Conclusion section has also been revised to ensure that the interpretation remains limited to the demonstrated nanoparticle fabrication, characterization, and antibacterial activity results.

**Location of revision:**

 Discussion section – Antibacterial mechanism paragraph; Conclusion section.

**Minor Comments**

**Comment:**

**Correct grammatical and typographical errors.**

**Response:**

 The manuscript has been carefully revised to correct grammatical errors, typographical mistakes, and improve overall language consistency throughout the manuscript.

**Comment:**

**Italicize bacterial species names consistently.**

**Response:**

 All bacterial species names have been reviewed and formatted according to microbiological nomenclature guidelines. The genus and species names are now consistently italicized throughout the manuscript.

**Comment:**

**Improve figure quality.**

**Response:**

 We appreciate the reviewer’s suggestion regarding figure quality. We have carefully reviewed all figures in the manuscript and ensured that they are presented with the highest available resolution from the original experimental data. The figures were checked for clarity, labeling accuracy, and consistency with the corresponding descriptions in the text.

 Due to the limitations of the original image acquisition files, further enhancement of the figure resolution was not possible without affecting the original data representation. Therefore, the figures have been retained in their original form to preserve the accuracy and integrity of the experimental results.

**Comment:**

**Update several references with recent literature.**

**Response:**

 Several references have been updated, and recent literature has been incorporated to strengthen the scientific background and discussion regarding PLGA nanoparticles, green synthesis approaches, and antibacterial applications.

**Final Statement**

 We sincerely thank the reviewer for the valuable comments and constructive suggestions. The revisions have improved the scientific accuracy, clarity, and overall quality of the manuscript.

# Reviewer report: Reviewer response for version 1

**DOI:** 10.5256/f1000research.191530.r511839

**Published:** 2026-09-10

**Authors:** Waseem Ahmad

**Affiliations:** 1 Graphic Era (Deemed to be University), Dehradun, India

1. Explain the reason behind selecting *Phoenix dactylifera L seed extract* for this synthesis. 

 2.Please clarify the innovations of this work in the introduction section.

 3.Characterization apparatus and write-up should be re-checked.

 4. plz mention the lattice plane in the XRD spectra 

 5.Plz Produce the UV spectra also

 6. Plz provide the magnification of SEM images

Is the work clearly and accurately presented and does it cite the current literature?

Partly

If applicable, is the statistical analysis and its interpretation appropriate?

Partly

Are all the source data underlying the results available to ensure full reproducibility?

Yes

Is the study design appropriate and is the work technically sound?

Yes

Are the conclusions drawn adequately supported by the results?

Partly

Are sufficient details of methods and analysis provided to allow replication by others?

Partly

Reviewer Expertise:

Green synthesis of nanoparticles and their application in waste water treatment.

I confirm that I have read this submission and believe that I have an appropriate level of expertise to confirm that it is of an acceptable scientific standard, however I have significant reservations, as outlined above.

# Response

**Published:** 2026-09-19

**Authors:** Elaf Sameer

**Affiliations:** Biology, Mustansiriyah University Department of Biology, Baghdad, Baghdad Governorate, Iraq

Response to Reviewer 2

 Comment 1: Explain the reason behind selecting Phoenix dactylifera L. seed extract for this synthesis.

 Response: Thank you for this valuable comment. The rationale for selecting Phoenix dactylifera L. seed extract has been clarified in the revised Introduction section. Date seeds were selected because they represent an abundant agricultural by-product containing naturally occurring phytochemical compounds. Their utilization provides a sustainable approach by converting agricultural waste into a value-added component during PLGA nanoparticle fabrication. The extract was used as a natural component during synthesis, while the contribution of individual extract constituents requires further comparative investigation.

 Comment 2: Please clarify the innovations of this work in the Introduction section.

 Response: Thank you for this suggestion. The novelty of the study has been clarified in the Introduction section. The revised manuscript highlights the green synthesis of PLGA nanoparticles using Phoenix dactylifera L. seed extract as a sustainable biological component and the evaluation of the developed nanoparticles against clinical osteomyelitis-associated bacterial isolates.

 Comment 3: Characterization apparatus and write-up should be re-checked.

 Response: The characterization section has been revised by adding the details of the instruments used. FE-SEM was performed using TESCAN MIRA3 (Czechia), XRD using PHILIPS PW1730 (The Netherlands), and FTIR using PerkinElmer TWO (USA).

 Comment 4: Please mention the lattice plane in the XRD spectra.

 Response: The XRD pattern was re-evaluated. Since the prepared PLGA nanoparticles exhibited broad diffraction features associated with an amorphous polymeric structure, no distinct crystalline diffraction peaks were observed; therefore, specific lattice planes (hkl) could not be assigned. This clarification has been added to the revised manuscript.

 Comment 5: Please produce the UV spectra also.

 Response: Thank you for this suggestion. UV-Vis spectroscopy was not included in the original experimental design and was therefore not available for the current study. This limitation has been acknowledged, and UV-Vis analysis is suggested for future investigations.

 Comment 6: Please provide the magnification of SEM images.

 Response: The magnification information available on the SEM micrograph has been added to the revised figure caption. The SEM image of PLGA nanoparticles is presented with a magnification of 140,000× and a scale bar of 500 nm.

 Thank you again for the constructive comments. All suggestions have been carefully considered and incorporated into the revised manuscript to improve clarity, reproducibility, and scientific presentation.
